# Denoising of Fluorescence Lifetime Imaging Data via Principal Component Analysis

**DOI:** 10.21203/rs.3.rs-7143126/v1

**Published:** 2025-07-29

**Authors:** Soheil Soltani, Jack G. Paulson, Emma Fong, Shannon M. Mumenthaler, Andrea M. Armani

**Affiliations:** 1 Ellison Medical Institute, Los Angeles, California 90064, USA; 2 Mork Family Department of Chemical Engineering and Materials Science, Viterbi School of Engineering, University of Southern California, Los Angeles, California 90089, USA; 3 Alfred E. Mann Department of Biomedical Engineering, Viterbi School of Engineering, University of Southern California, Los Angeles, California 90089, USA; 4 Ming Hsieh Department of Electrical and Computer Engineering – Electrophysics, Viterbi School of Engineering, University of Southern California, Los Angeles, California 90089, USA

**Keywords:** principal component analysis, FLIM, Fluorescence Lifetime Imaging Microscopy, Denoising, Organoid imaging, FLIM analysis, metabolic imaging, adaptive noise removal, data retention

## Abstract

Fluorescence Lifetime Imaging Microscopy (FLIM) quantifies autofluorescence lifetime to assess cellular metabolism, therapeutic efficacy, and disease progression. These dynamic and heterogeneous processes complicate signal analysis. Fit-free analysis methods such as phasor analysis are increasingly used due to limitations of fit-based approaches. However, incorporating photon-counting shot noise often leads to moderate-to-high uncertainty in detecting subtle changes. Common noise-reduction strategies can introduce errors and cause data loss. We developed noise-corrected principal component analysis (NC-PCA), which selectively identifies and removes noise to isolate the signal of interest. We validated NC-PCA by analyzing FLIM images of patient-derived colorectal cancer organoids treated with various therapeutics. First, we show NC-PCA decreases uncertainty by up to 5.5-fold compared to conventional analysis and reduces data loss over 50-fold. Then, using a merged dataset, NC-PCA reveals multiple metabolic states. Overall, NC-PCA offers a powerful, generalizable tool to enhance FLIM analysis and improve detection of biologically relevant metabolic changes.

## Introduction

Fluorescent Lifetime Imaging Microscopy (FLIM) is a versatile and real-time imaging method used across cell biology, neuroscience, and cancer research^1–3^. This broad impact is due to its ability to reveal information across multiple length scales, allowing dynamic biological processes, cellular structures, and molecular interactions to be measured^4,5^. Over the past several decades, numerous variations of FLIM have been developed by leveraging advances in signal processing, automation, and photodetectors. Unlike conventional imaging which detects the intensity of a fluorescent signal, FLIM analyzes the fluorescent lifetime. In the context of biology, changes in fluorescent lifetimes can be correlated to protein-protein interactions^4,5^, fluctuations in pH or temperature^6–8^, and molecular dynamics^9,10^. Moreover, FLIM improves the detection of the intrinsic autofluorescence signal that is characteristic of cellular metabolism, enabling fundamental cell biology investigations and advancing therapeutic development^11–13^.

Originally, FLIM analysis leveraged exponential fitting methods to extract fluorescent lifetimes, where a linear combination of exponentials was used to fit per-pixel intensity decays^14–16^. While this approach can quantitatively determine the lifetimes and contribution fractions in large datasets, it has high computational costs and high susceptibility to noise resulting in inaccuracies or even convergence errors. These shortcomings have led to the development of fit-free analysis methods such as phasor analysis. This strategy translates FLIM data into Fourier space, mapping the per-pixel intensity decay onto orthogonal vectors^16,17^. Any combination of these components corresponds directly to the pixels and represents a unique lifetime combination. This approach can reveal single or multiple dominating lifetimes through clustering in distinct regions on the phasor histogram plot^16,18^ with reduced computational load. However, transformations into phasor space inherently integrate the intrinsic counting noise which complicates the identification of distinct clusters and subtle shifts of phasors^19^.

**Figure 1.**
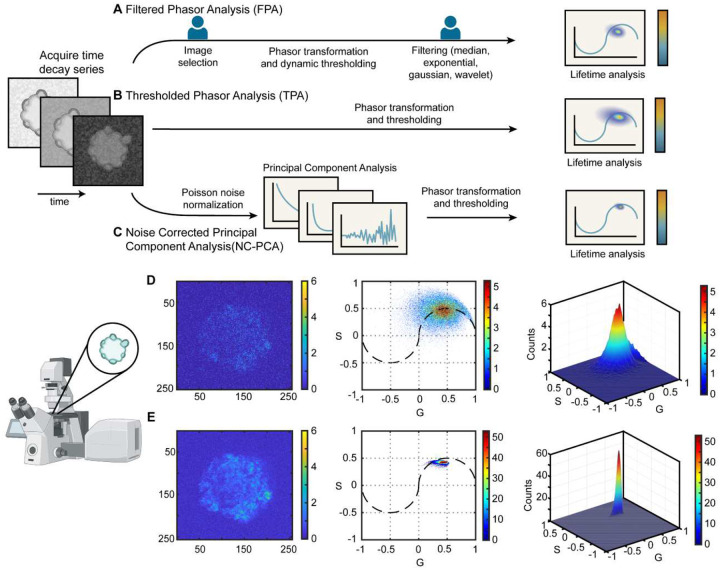
Phasor analysis workflows for FLIM data. After image acquisition on a FLIM system, the data is analyzed using different approaches. (**A**) Filtered phasor analysis method (FPA) includes image selection based on total counts, dynamic thresholding, and application of a selective smoothing filter. (**B**) Thresholded phasor analysis (TPA) only involves the application of an adaptive threshold. (**C**) Noise-corrected Principal Component Analysis (NC-PCA) begins with a Poisson noise normalization, then application of PCA, and subsequent thresholding and phasor transformation. (**D**) Example of results obtained using the TPA analysis method. First time-bin image of a patient-derived colorectal cancer organoid, 2D phasor histogram, and 3D phasor histogram. (**E**) Example of results obtained using the NC-PCA method. Note the reduction in S and G spread in the NC-PCA phasor plot and the 10x difference in counts between parts (**D**) and (**E**) as a result of the improved phasor clustering.

Common remedies for noise sensitivity include intensity thresholding (TPA) and a filter-based (FPA) phasor analysis^19,20^ (Figures 1A, 1B, S1, and S2). While effective for small datasets with low-noise signals, these methods have two main limitations. First, for larger datasets, adaptive thresholding is required to account for varying noise levels. Second, the common smoothing filters, such as median, exponential, and Gaussian, are susceptible to introducing smoothing errors^21^. Therefore, frequent filter parameter tuning is required. Other noise reduction techniques such as wavelet filtering have shown superior denoising capabilities but involve complexities like wavelet bias selection, parameter tuning, and leakage between bands^22,23^. More recently, machine learning models are being explored as a means of noise reduction^19^. However, these models depend heavily on high-quality, labeled training data for accuracy, which is hard to acquire and is often missing in rapidly emerging fields.

An alternative for analyzing FLIM datasets is a dimensionality reduction method known as Principal Component Analysis (PCA). This technique selectively removes noise while retaining structured data through a transformation into a new orthogonal basis set^24,25^, constructed by the eigenvectors and eigenvalues of a mean-centered covariance matrix. These vectors, or principal axes, are sorted in decreasing variance order. Thus, PCA enables precise identification of important features with highest variance and facilitates effective removal of noise in complex datasets. Additionally, because it is a data-driven denoising scheme and it does not rely on a priori knowledge of the system^26^, it removes potential bias. As a result, PCA has been effectively applied in a diverse array of fields, including data and image compression, omics research, and financial analysis^27–29^.

In the present work, we develop a noise-corrected-PCA (NC-PCA) method for denoising FLIM data and validate the approach using both simulated and experimental images (Figure 1C). Our NC-PCA analysis technique simultaneously denoises time-domain FLIM signals and enhances phasor domain accuracy (Figure 1D and 1E) by reducing Shot noise and preserving the correlated linearity of corresponding pixels^27,30–32^. To confirm the method, we created and analyzed synthetic FLIM data as well as a standard fluorescent dye with known ground truth values. Next, we used the NC-PCA method to analyze patient-derived colorectal cancer spheroids and confirmed the improvement in performance as compared to the TPA approach. We showed that data retention is improved by ~50-fold and the uncertainty is reduced by ~5-fold. Finally, we analyzed the metabolic activity of patient-derived colorectal cancer organoids in response to standard therapeutics using all three approaches in Figure 1. In comparison to TPA and FPA, NC-PCA demonstrated a reduction in error as high as ~58% and ~45%, respectively. Finally, using an overlaid dataset consisting of untreated and treated organoids, we demonstrated that NC-PCA can resolve multiple emission lifetimes which are indicative of distinctly different metabolic states.

## Results

### Validation of NC-PCA using synthetic data

**Figure 2.**
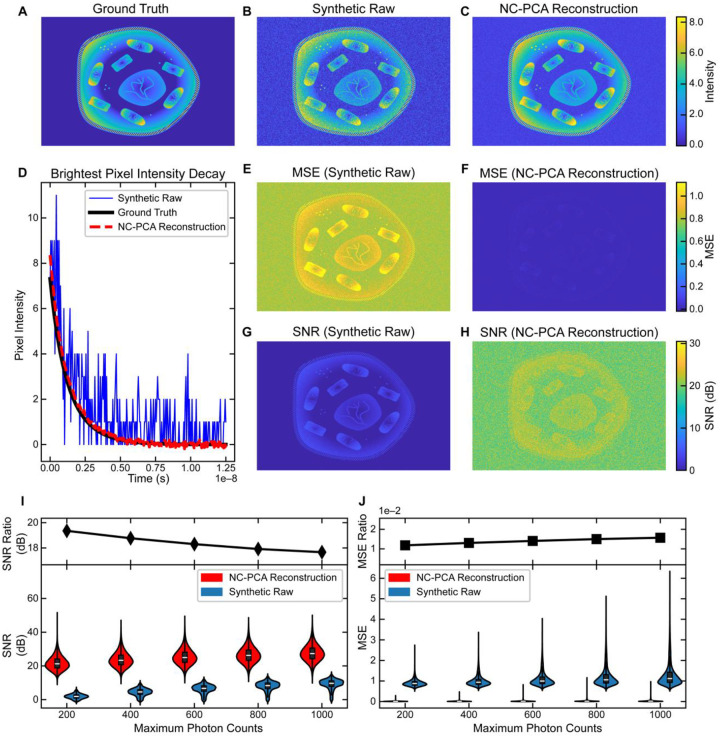
Ground truth comparison of NC-PCA on synthetic FLIM data. The first time bin of the synthetically created data that serves as the (**A**) ground truth and (**B**) that is modified with Poisson noise to serve as the synthetic raw data. A range of photon count levels were investigated, and this data is limited to 200 photon counts, representing extremely noisy data. (**C**) The image shown in part (**B**) is reconstructed using NC-PCA. (**D**) The intensity of the brightest pixel in the ground truth image (solid black), synthetic raw data (solid blue), the NC-PCA (red dashed). (**E, F**) MSE and (**G, H**) SNR are calculated for each pixel across the entire synthetic raw image or NC-PCA reconstructed image. (**I, J**) To explore the role of noise in reconstruction ability, the photon counts were varied from 80 photon counts (low SNR) to 1800 photon counts (High SNR). All results are included in the SI, and a subset of the results are presented. The violin plots for (**I**) SNR and (**J**) MSE images from synthetic raw and NC-PCA reconstruction images highlighting the improvement offered by NC-PCA, in terms of data spread and relative SNR and MSE values. NC-PCA improvement was quantified via SNR and MSE Ratios between synthetic raw and NC-PCA reconstruction.

To demonstrate the effectiveness of NC-PCA for FLIM noise reduction, we generated a synthetic time-series dataset based on a 540×720 pixel image of a cell. The image included distinct geometric features representing cellular structures such as the nucleus, mitochondria, vacuoles, ribosomes, and cell wall. Each nonzero pixel was assigned a fluorescence lifetime that governed its exponential decay. Using these lifetimes, we simulated a 256-frame time-series with pixel intensities decaying over time to model fluorescence lifetime behavior. This dataset served as a ground truth reference for evaluating NC-PCA’s reconstruction and denoising accuracy. Figure 2A shows the first time bin of the ground truth.

The noise distribution present in FLIM data is primarily Poisson noise, commonly referred to as Shot noise, where noise arises from counting errors with a standard deviation proportional to the square root of photon counts^32^. To simulate this, we applied a randomized Poisson distribution to each pixel, using the corresponding time-bin photon count as the mean value. For background pixels with no assigned lifetime, a mean of 0.8 was used to ensure nonzero noise, based on experimental results (see SI).

Since the signal-to-noise ratio (SNR) of Shot noise scales with square root of photon counts, we varied the noise characteristics by adjusting the total photon counts for every decay signal per pixel. The range of total counts per image was chosen to reflect observed values in experimental datasets with representative photon counts ranging from 200 to 1000 photons. This approach ensured that our calculated noise accurately modeled the variations encountered in experimental FLIM data. Additional details are provided in the SI.

As a representative example, Figures 2B and 2C show the first time bin of the synthetic data with 200 photon counts and the corresponding NC-PCA reconstruction. Signal-to-noise ratio (SNR) and mean square error (MSE) are complementary metrics for evaluating reconstruction quality. Higher SNR reflects more robust reconstruction; lower MSE indicates greater similarity. Both metrics were computed for the raw and NC-PCA reconstructed images by analyzing pixel intensity values across all time bins.

Figure 2D highlights the decay of the highest intensity pixel, revealing substantial signal quality improvements after NC-PCA reconstruction. The SNR increased nearly threefold, from 5.99 dB in the synthetic image to 17.5 dB post-reconstruction. Similarly, MSE dropped over an order of magnitude, from 1.34 to 0.05. These results underscore NC-PCA’s ability to enhance signal fidelity, especially in low-photon-count pixels. This ability will directly advance applications where data acquisition speed is a priority, such as monitoring dynamic biological systems.

Similar analysis can be performed for the entire dataset which encompasses 388,800 pixels over 256 time bins. The results from this spatial analysis are detailed in Figure 2E–2H. Figure 2E and 2F display the SNR before and after NC-PCA reconstruction while Figure 2G and 2H present the corresponding MSE. To allow direct comparison the scale bars are unified in these figures. Quantitatively, the median SNR in the synthetic dataset Figure 2E is 1.87 dB, which improves to 21.2 dB post NC-PCA reconstruction (Figure 2F). In parallel, the median MSE decreases from 0.875 in Figure 2G to 0.0101 after NC-PCA reconstruction (Figure 2H). Taken together, NC-PCA results in nearly a ~20 dB improvement in SNR and ~90x improvement in MSE.

To further evaluate NC-PCA in FLIM analysis, we assessed improvements in SNR and MSE as photon counts increased from 200 to 1000. Increasing photon counts in the model serves as a proxy for increasing frame number in an experiment. Examples of synthetic images with noise levels across this range, their NC-PCA reconstructed counterparts, and the corresponding MSE and SNR maps are provided in Figure S7. The distributions of SNR and MSE at each photon count level are shown in the violin plots in Figure 2I and 2J. Across the entire range studied, NC-PCA improved both SNR and MSE. These findings underscore the ability of NC-PCA to enhance signal fidelity specially in low photon count pixels.

In addition, we define two metrics to quantify NC-PCA improvement: the SNR ratio and MSE ratio. The former, expressed in decibels, is the difference between the median SNR of the NC-PCA reconstruction and the noise-embedded synthetic data. The latter is the median MSE of the NC-PCA reconstruction divided by that of the noise-embedded synthetic data. As shown in Figures 2I and 2J, both metrics indicate clear improvement. As photon counts increase, the noise-embedded image SNR improves, bringing decay signals closer to the ground truth. Thus, while NC-PCA consistently enhances SNR and MSE, the degree of improvement lessens at higher photon counts.

### Validation of NC-PCA approach using experimental FLIM data

When imaging dynamic biological systems using FLIM, the number of acquired frames is often lowered to maintain acceptable image acquisition rates. For example, the number of acquired frames in our system is normally within the range of 10–30 frames which allows the minimum total counts per pixel to be greater than 100. By increasing the number of acquired frames to ~100–200 frames, the total photon count increases to 7,000–10,000, and the accuracy of the data analysis improves. However, this increase in frame number dramatically increases the image acquisition time up to 10-fold, which negatively impacts the ability to capture dynamic information.

To confirm that NC-PCA can reliably reconstruct a low frame or low photon count dataset, a solution containing a single fluorophore, Coumarin-6, was imaged with two frame acquisition values: 30 and 100. All other conditions, including sample preparation and microscope settings, were identical between the measurements. The decrease in noise during the 100-frame measurement allowed this data to serve as ground truth. To obtain lifetime values, a single component exponential fit was applied to every pixel in each image. In addition, NC-PCA was performed on the 30-frame acquisition data. Prior work has demonstrated that Coumarin-6 exhibits a single fluorescence decay pathway with lifetime values in the range of 2.43–2.60 ns^33,34^. Because only one lifetime is present, the phasor distribution should be located on the unit circle, and this fluorophore provides an ideal experimental testbed to evaluate the NC-PCA method.

**Figure 3.**
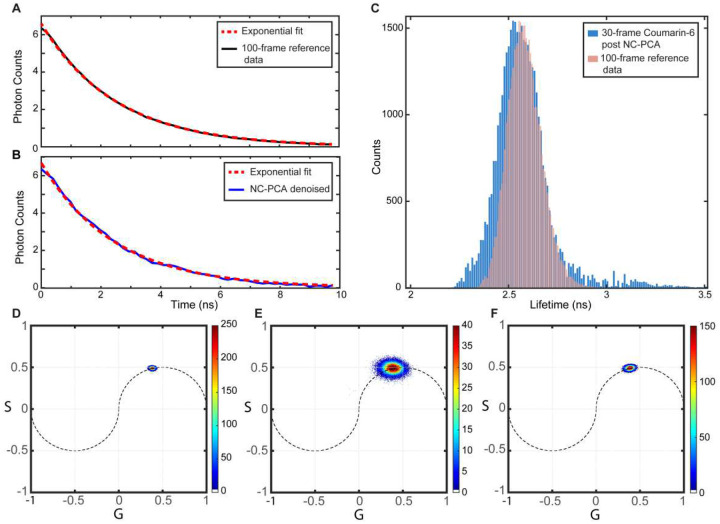
Comparison of lifetime distribution between the 100-frame ground truth FLIM signal and 30-frame raw data post NC-PCA reconstruction for Coumarin-6. Representative temporal decay FLIM signal overlaid on single exponential fit for (**A**) 100 frame ground truth (**B**) 30 frame raw data post PCA reconstruction. (**C**) comparison of histograms for extracted lifetimes from 100-frame ground truth and post NC-PCA denoising for 30-frame Coumarin-6 FLIM images. Corresponding phasor histograms for (**D**) 100-frame ground truth (**E**) 30- frame raw data (**F**) 30-frame raw data post NC-PCA reconstruction.

Figure 3A presents a representative temporal intensity decay for the 100-frame ground truth data that is fit to a single exponential decay. Attempts to fit the raw 30-frame data were unsuccessful because they either did not converge or resulted in large error due to insufficient signal-to-noise ratios. However, after applying NC-PCA to the 30-frame data, the previously obscured signal became clearly identifiable, and the data could be reliably fit (Figure 3B). Thus, the subsequent discussion focuses on a comparison between the 100-frame ground truth data and the NC-PCA analyzed 30-frame data.

Figure 3C shows the lifetime distribution from the ground truth image and from the NC-PCA reconstructed 30-frame FLIM image. The histogram of ground truth values has a mean of 2.58 ns and a Full Width Half Maximum (FWHM) of 0.2 ns. In comparison, the histogram of the NC-PCA constructed 30-frame data yields a mean lifetime of 2.56 ns and a FWHM of 0.23 ns. Both values fall are in agreement with prior results and are self-consistent^33,34^

In addition, we have calculated the phasor histograms for the 100-frame ground truth, raw 30-frame, and PCA reconstructed Coumarin-6 images. The results are shown in Figure 3D–3F. To quantitatively assess the data spread, the FWHM along the G axes is calculated for each dataset. The FWHM in the data significantly increases from 0.061 to 0.2217 when the frame rate decreases from 100-frame to 30-frame (Figure 3D and 3E). However, when NC-PCA is applied to the 30-frame data, the FWHM is reduced to 0.086, which is comparable to the 100-frame ground truth. Thus, NC-PCA effectively reduces the noise content in the raw low photon data, reducing the uncertainty in phasor histogram. These results demonstrate that NC-PCA allows 30frame data to achieve a similar fidelity to the 100-frame ground truth, enabling faster acquisition rates with minimal degradation in accuracy.

### Validation of NC-PCA approach using patient-derived colorectal cancer spheroids

FLIM imaging is routinely used to evaluate a biological system’s response to a perturbation by performing a phasor transformation of FLIM data and analyzing two variables, G and S. Together, these parameters represent the position and direction of shifts on the phasor histogram. The shifts reveal information regarding the fluorescent lifetime of different components in a sample, which can be correlated to changes in metabolic activity^11,13,35,36^.

When a single lifetime is present, G and S fall directly on the universal unit circle, as with Coumarin-6 (Figure 3). Shorter lifetimes lie near the edge (G=1, S=0), while longer lifetimes are closer to the origin. Points inside the unit circle represent weighted sums of lifetimes on the circle. Therefore, the phasor histogram position reflects the sample’s exact lifetime composition. However, decay signal noise in each pixel broadens the histogram and limits measurement accuracy^15,37^.

**Figure 4.**
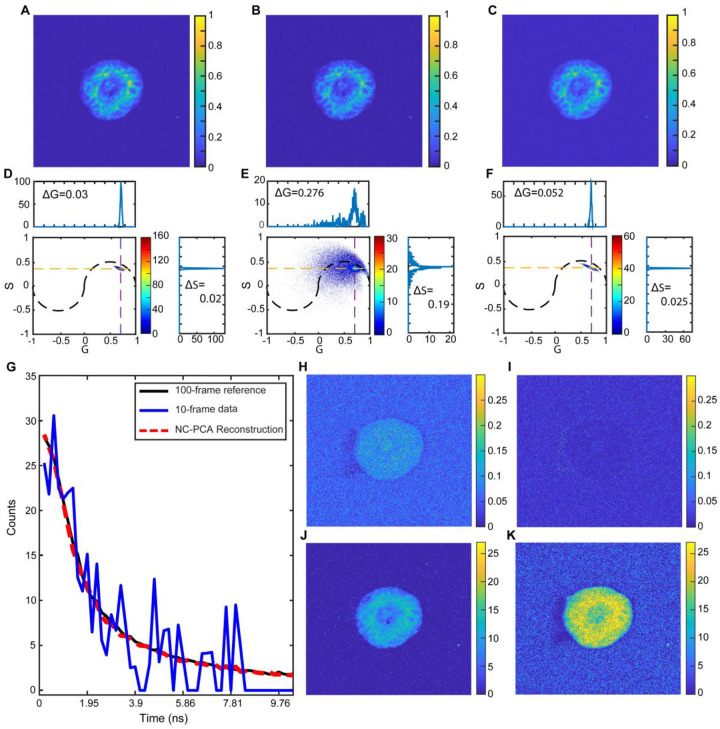
Application of NC-PCA on a patient-derived colorectal cancer organoid. FLIM images of (**A**) 100-frame FLIM data used as low-noise reference data, (**B**) 10-frame FLIM data, and (**C**) NC-PCA denoised 10-frame FLIM data. (**D-F**) Phasor plot of (**D**) reference data, (**E**) 10-frame FLIM data, and (**F**) NC-PCA denoised 10-frame FLIM data. The median count coordinates and the FWHM along the G and S axes are calculated along the dashed lines (**G**) The intensity of the brightest pixel in the reference image (solid black), 10-frame image (solid blue), and NC-reconstruction (red dashed) tracked over time. (**H, I**) MSE and (**J, K**) SNR are calculated for each pixel across the entire (**H, J**) 10-frame image and (**I, K**) NC-PCA reconstructed image using the 100-frame data set as the reference.

The ability of NC-PCA to overcome this limitation is demonstrated by temporally denoising FLIM signals from patient-derived colorectal cancer organoids. Similar to the previous measurement, we used 100-frame data as the ground truth and 10-frame imaging with and without NC-PCA for the experimental measurement confirming relevancy to biological applications. All other conditions, including sample preparation and microscope settings, were identical between the measurements. The first time-bin images for all three measurements are shown in Figure 4A–4C, and the associated phasor plots are in Figure 4D–4F.

Qualitatively, the G and S data, cluster around similar values across all three phasor plots; however, the 10-frame dataset shows a significantly broader distribution.; To quantify the similarities and differences, we calculated the median count coordinates and FWHM along the G and S axes (Figure 4D–4F). The NC-PCA denoised 10-frame data differs from the 100-frame ground truth by less than 0.3% in G and 0.25% in S (ground truth median coordinates: (0.7103, 0.3502)). Additionally, the FWHM of the NC-PCA reconstruction is nearly 5x narrower than the initial 10-frame dataset and is closely comparable to the ground truth FWHM.(FWHM for the 10-frame data is 0.276 and reduces to 0.052 after NC-PCA denoising, this value is comparable to the FWHM of the ground truth which is ~0.03). Therefore, NC-PCA reduces the data uncertainty while maintaining the median values.

The quality of the reconstruction was further quantified by calculating the SNR and MSE at the single pixel level (Figure 4G) and across the entire dataset (Figure 4H–4K) using the 100-frame dataset as the ground truth. For the given pixel, the SNR increased by 9.7dB, and the MSE decreases by ~9.4 when the NC-PCA method is applied to the 10-frame dataset. When the entire dataset is evaluated, similar trends in SNR (Figure 4H and 4I) and MSE (Figure 4J and 4K) are observed. We find an average improvement in the SNR by ~10 dB and a decrease in MSE by ~10 fold.

Additionally, NC-PCA reconstruction adaptively denoises the low frame FLIM signal, resulting in an improvement in the time domain data and consequently, in the fit-free phasor domains. This improvement significantly enhances the phasor domain detection accuracy with no information lost during the process. Notably, the phasor domain detection resolution improved from 0.276 to 0.052 even as the frame number decreased from 100- to 10-frames.

### Assessment of Data Loss

One application of FLIM imaging is evaluating metabolic changes in biological systems. In combination with 3D cultures, this method is frequently used as part of a therapeutic development pipeline. For the specific case of imaging cellular metabolism, the change in autofluorescent emission of NADH as it shifts between free and protein-bound states is often leveraged^12,36,38^. Because these two states have distinctly different autofluorescent lifetimes, it is possible to detect metabolic changes by monitoring the phasor histogram. Typically, the results are reported in terms of the fraction bound (fB) NADH, which is directly proportional to G and S (Figure S4 and S5).

Like many biological imaging measurements, these metabolic FLIM datasets can suffer from significant variable noise, integrated directly into G and S. In efforts to simplify the interpretation and increase statistical confidence, the phasor distribution is often collapsed into a single pair of S and G values post-filtering. This technique requires that the entire phasor distribution of each image is averaged, and the histogram state of the image is then approximated by a single pair of values. This approach simplifies the phasor plot and may lead to a loss of information. NC-PCA provides a path to reduce the impact of detection noise while maintaining the complexity of the lifetime data, improving data analysis rigor.

**Figure 5.**
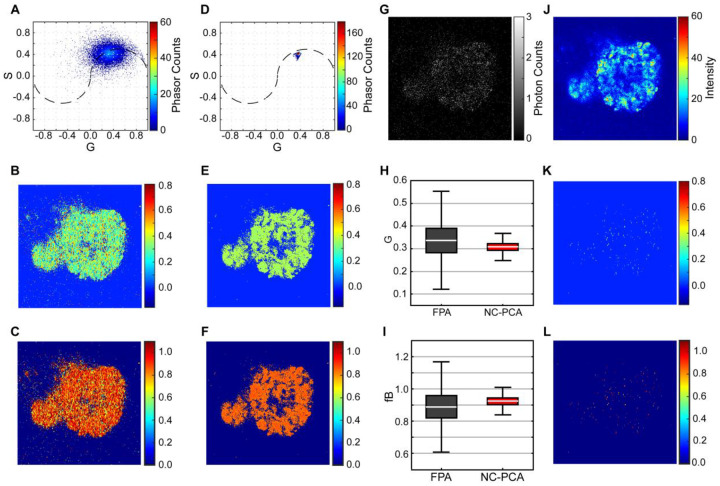
Comparison of phasor distributions of a spheroid using filtered phasor analysis and NC-PCA analysis. Phasor histogram, G, and fraction bound images of a spheroid analyzed using (**A-C**) filtered phasor analysis and (**D-F**) NC-PCA analysis. (**G**) Grey scale image of the first plane photon count. (**H, I**) comparison of boxplots for G and fraction bound distributions using FPA and NC-PCA phasor analysis methods. (**J**) Total photon count per pixel of the same FLIM image used in (**A**). Pixel maps of G (**K**) and fraction bound (**L**) images highlighting the pixels that preserve their original values if the entire phasor histogram is approximated by average G and S values (data preservation map).

To evaluate the impact of post-filter averaging and demonstrate the capability of NC-PCA to obviate the need for single-point approximation, we calculated the phasor histograms of a representative image using either the FPA or NC-PCA phasor histogram methods. Figure 5A–5C presents the phasor histogram, G, and fB images, respectively, of the FLIM data processed using the FPA process outlined in Figure 1A. Figure 5D–5F shows the same image processed using the NC-PCA method (see SI for details).

While applying a median filter helps localize the phasor histogram and smooths the G and fB images, the resulting distributions still display significant spread (Figure 5A–5C). This ambiguity highlights the limitation of the median filter in denoising and necessitates aggressive thresholding, filtering, and subsequent phasor averaging. In contrast, the NC-PCA method effectively denoises the phasor histogram, as well as the G and fB images, reducing the uncertainty and spread of phasor distribution (Figure 5D–5F). Therefore NC-PCA precludes the need for post-filter averaging.

The effective denoising capabilities of NC-PCA compared to FPA is further illustrated when the images obtained using both methods are compared. The G and fB images obtained from filtered phasor analysis (Figure 5B and 5C) closely resemble the first time-bin image which is a noisy representative of the FLIM image (Figure 5G). In contrast, the G and fB images derived from NC-PCA (Figure 5E and 5F) more closely correspond to the total count map (Figure 5J), an aggressively filtered representation of the FLIM data. This comparison also demonstrates that NC-PCA is a more potent denoising method compared to FPA.

The reduction in the phasor distribution using NC-PCA is also quantitatively illustrated in Figure 5H and 5I where the boxplots of G and fB are shown. There is a ~5-fold ratio between the standard deviation of the G component and fB distributions using NC-PCA compared to FPA method. This highlights the limitation of FPA method in denoising and necessitates frequent manual adjustment of filter parameters and continuous evaluation for data loss, underscoring its limited utility compared to NC-PCA.

To show the degree of data loss incurred through the FPA process, we have calculated the map of the G and fB values that maintain their original values in case the phasor histogram is averaged and approximated by a single pair of G and S. As clearly highlighted in Figure 5K and 5L, less than 2% of data points in G and fB maps are preserved throughout averaging post the FPA process, emphasizing the fact that averaging the FLIM histogram can result in over smoothing and significant data loss. In contrast, NC-PCA effectively denoises the FLIM data such that the uncertainty in phasor histogram reduces to a degree that averaging is no longer is required and averaging errors are obviated. As a result, the data is preserved when NC-PCA is used.

### Revealing Metabolic Dynamics and Improved Resolution of Drug Effects

**Figure 6.**
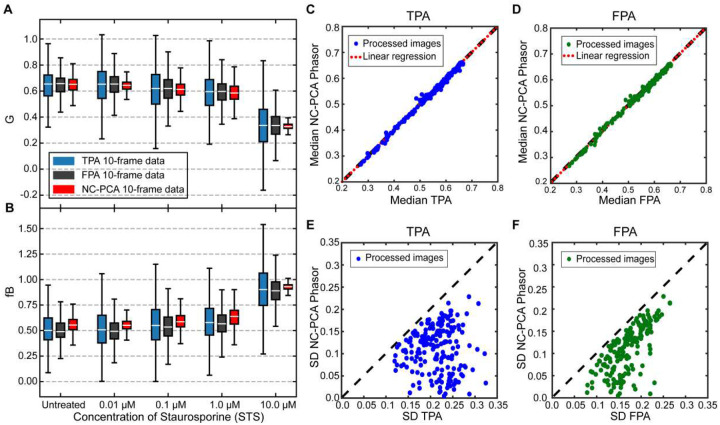
Statistical analysis of phasor distributions with and without NC-PCA. (**A**) Boxplot of G component of phasors using TPA (blue), FPA (grey), and NC-PCA denoised data (red) for untreated, 0.01, 0.1, 1, and 10.0 μM of STS. (**B**) Box plots of fraction bound values using TPA (blue), FPA (grey) and phasor analysis of the same data after NC-PCA denoising (red), (**C, D**) Comparison of median values for G between NC-PCA vs FPA and TPA respectively. The y=x (black dash) and linear regression fit (red dotted line) are shown for comparison. (**E, F**) Comparison of standard deviation for G values between NC-PCA vs FPA and TPA respectively. As a reference, the line y=x is shown in black dash.

While detecting extreme metabolic responses confirms that a response occurs, fully understanding a physiological system requires mapping the entire dose-response curve. To demonstrate NC-PCA’s capability for such analysis, we used an existing dataset of patient-derived colorectal cancer organoids treated with various therapeutics (see Table 1) along with a control solution (untreated)^36^. The total dataset consisted of 207 total images with over 6 million data points. The results for one therapeutic are shown in Figure 6, and additional analysis for all therapeutics and concentrations studied are in the SI (Figure S9–S13). A summary of all experiments is shown in Table 1.

A cursory inspection of Figure 6A and 6B reveals one advantage of NC-PCA. TPA or FPA methods can result in spread or even skewed phasors that have data that is outside physically realizable values due to the incorporation of noise. For example, results outside the universal unit circle or fraction bound values above 1 or below 0 are not physically possible. As shown in Figure 6A and 6B, values calculated using NC-PCA-reconstruction fall within the biologically realizable range.

A key aspect of any denoising or filtering method is that it should preserve the information and the structure of the data. Based on the prior measurements, we expect that NC-PCA should have a negligible effect on the median values for single cluster phasor distributions and simultaneously reduce the noise-associated spread. In Figure 6A, it appears that NC-PCA does not significantly change the median values in G and fB. To quantitatively determine if the data median is preserved, we created a single pooled dataset using all the STS-treated organoids in all the conditions (size n=207 images), analyze it using TPA, FPA and NC-PCA phasor analysis methods, and evaluate the median values of G. The calculated slopes are 1.0097 and 0.998 for Figure 6C and 6D, respectively. A slope of 1 means that the x and y axis values are identical. Thus, these slope values confirm that the data is preserved across all experimental conditions when NC-PCA is used.

The overarching goal of the NC-PCA method is to reduce the error. As can be observed in Figure 6A and 6B, the NC-PCA-driven workflow results in a reduction in both the upper and lower quartiles as well as in whisker length across all STS concentrations studied. To evaluate this metric more broadly, the standard deviation (SD) of G of the pooled dataset was calculated. There is a notable decrease in SD for all datasets when NC-PCA is used (Figure 6E and 6F). Taken together, the results in Figure 6C–6F show that NC-PCA effectively preserves key information from the dataset while decreasing uncertainty.

Lastly, by comparing the SD values in Figure 6E and 6F, an interesting feature of the NC-PCA method, highlighted in Figure 6A, becomes more evident. The SD values calculated for the FPA method are smaller compared to TPA method; however, NC-PCA produces the smallest standard deviation among the methods. This signifies the fact that while filters can remove noise to certain extent, their ability is limited and is not as effective as the NC-PCA method. This fact proves that NC-PCA can efficiently denoise the data with no need for extreme thresholding nor truncation of lower ranked principal components.

**Figure 7.**
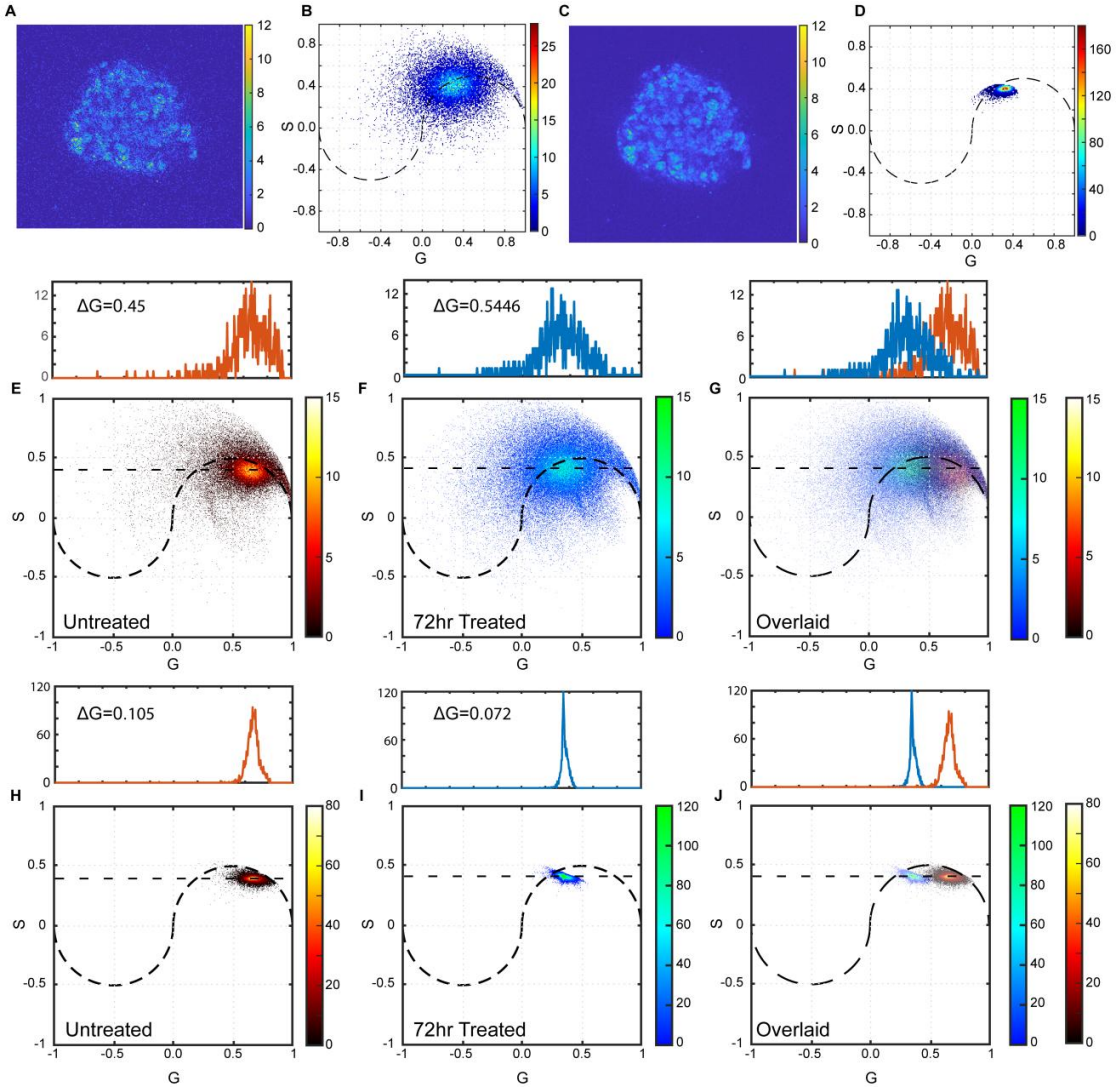
Detecting time dependent effects of STS on patient-derived colorectal cancer organoid metabolism. First time bin and corresponding phasor histogram of an untreated patient-derived colorectal cancer organoid data analyzed using (**A, B**) TPA approach or (**C, D**) NC-PCA approach. Data was taken using 10-frame acquisition. (**E**) Phasor histograms of untreated and (**F**) 72-hour, 10μM STS-treated organoids analyzed using the FPA approach. (**G**) The phasor histograms in parts (**E**) and (**F**) are overlaid. The count distributions along the maximum count lines are fit to Gaussian distributions. (**H, I**) The same data set is analyzed using the NC-PCA approach, and (**J**) the pair of phasor histograms are overlaid. The count distributions along the maximum count lines are fit to Gaussian distributions.

In the context of FLIM analysis, the minimum resolvable signal change on the phasor histogram is the FWHM or signal linewidth of the phasor accumulation point. Broad phasor distributions limit the ability to detect subtle metabolic responses within the same image. To demonstrate the ability of NC-PCA to overcome this challenge, a subset of the data in Figure 6 was used. Specifically, 10 datasets of untreated and 72hr, 10μM STS-treated organoids are calculated and then merged, creating an overlaid dataset that contains two known metabolic states. Figure 7A–7D show a representative first time-bin image and phasor histogram of a patient derived organoid processed using TPA and NC-PCA methods. Note that FPA is not a reversible process, and reconstruction of images is not possible. Therefore, reconstructions are limited to TPA and NC-PCA. Phasor plots of the whole dataset were analyzed (Figure 7E, 7F, 7H, and 7I) and subsequently overlaid (Figure 7G and 7J). In parallel, the histogram of the data along the G axis was calculated and is plotted as part of Figure 7E–7J.

As evident in Figure 7E and 7F, the two phasor distributions generated using FPA have significant overlap. Based on the FWHM of the associated histograms, the minimum detectable signal change on the histogram is limited to 0.5446. In Figure 7G, the separation distance between these histograms, defined as the difference in G-coordinates of their maximum counts, is ~0.31. This separation is nearly half the FWHM value. This analysis highlights why multiple metabolic states, or the metabolic heterogeneity within a single frame, is not distinguishable when FPA is used. This constraint arises from the limitation of the FPA process in denoising.

In contrast, NC-PCA effectively denoises the FLIM data and reduces the phasor histogram FWHM (Figure 7H–7J). As a result, while the separation between the two states is unchanged, the signal linewidth narrows to 0.105. This is ~5.5 times improvement in linewidth as compared to FPA method, and it is ~3 times smaller than the separation distance, making the two states fully resolvable. Importantly, the differences in the mean values of G as determined by both methods are not statistically significant, indicating that the NC-PCA method is not altering the data. Thus, NC-PCA can reveal multiple metabolic processes happening within a biological system that are not clearly distinguishable by conventional methods. Furthermore, NC-PCA can identify these distinct characteristics without the express need for extra smoothing and does not distort the underlying data.

Finally, to demonstrate that NC-PCA provides robust denoising independent of instrument, chemical treatment, or biological condition, we analyzed FLIM data acquired from two imaging systems (Zeiss and Olympus) using a range of therapeutics, therapeutic concentrations, and media composition (see Table S4). As shown in Figures S14–S15, the same improvement in noise that was observed in Figure 6, while retaining the median value, is observed again. These findings confirm effective noise suppression without altering signal dynamics and demonstrate the generalizability of NC-PCA.

## Discussion

Fluorescence Lifetime Imaging Microscopy (FLIM) is an important tool for studying complex biological phenomena, but its effectiveness is often hindered by noise, acquisition time, and photodamage. One of the primary challenges of FLIM analysis is achieving high signal fidelity while minimizing these limiting factors. An effective analytical tool should not only preserve data fidelity, but also adaptively suppress noise, particularly when photon counts are low.

Here, we demonstrate that NC-PCA effectively mitigates noise distortion while preserving the underlying signal in time-series FLIM data. A key advantage of this method is its adaptivity through data-driven decomposition, allowing it to handle large FLIM datasets efficiently without extensive manual intervention. This streamlining makes NC-PCA ideal for high-throughput FLIM studies. Furthermore, by reducing reliance on predefined settings, NC-PCA adjusts to varying data characteristics, ensuring consistent performance and enhancing reproducibility across different datasets. This makes NC-PCA a powerful tool for large-scale FLIM studies.

In addition, NC-PCA significantly broadens the scope of analytical operations that were previously limited to high photon count datasets. Using patient-derived colorectal cancer organoids, we demonstrated an average 10 dB improvement in SNR and a 10-fold reduction in MSE, highlighting NC-PCA’s effectiveness in improving data fidelity. By enhancing the SNR in low photon-count FLIM images, NC-PCA facilitates the efficient and accurate application of advanced techniques such as reference-free calibration, multi-exponential lifetime fitting, fluorescence lifetime-resolved anisotropy, Förster Resonance Energy Transfer (FRET) analysis, and machine-learning-based FLIM analysis^35,36,39,40^.

One major challenge in fluorescence imaging is balancing SNR against photodamage risk. Intense or prolonged laser exposure can generate reactive oxygen species, disrupt cellular function, and degrade fluorophores, limiting imaging duration and reliability.^41,42^. NC-PCA mitigates these issues by increasing the SNR up to 20 dB in datasets even under low photon-count conditions, enabling reduced laser power and shorter acquisition times without compromising data quality. This preserves fluorophore integrity, minimizes photodamage, and enables repeated or long-term measurements essential for tracking dynamic processes like stem cell differentiation, drug response, or tumor progression.^40,43,44^.

Taken together, these findings establish NC-PCA as a powerful and enabling tool for both fundamental biological research and translational applications. Its ability to extract high-fidelity lifetime data from low photon-count FLIM images enhances precision in detecting metabolic states, disease progression, and therapeutic response, broadening its impact in biomedical research^36,40,43–47^.

## Methods

### Data Analysis Methods

The FLIM data was analyzed using TPA, FPA, and NC-PCA through custom Python and MATLAB scripts. Notably, both scripts operate independently and produce identical results. Both the Python and MATLAB scripts are freely available on Zenodo (https://doi.org/10.5281/zenodo.14895223). While both scripts primarily process raw time-series TIFF files, they are designed for customization and adaptability. The algorithm implementation in both Python and MATLAB consists of three main components: a data preprocessing script, an NC-PCA analysis script, and a phasor analysis script. Adapting non-TIFF file formats for compatibility with the NC-PCA and phasor analysis scripts simply requires converting the imported data into arrays.

The TPA workflow is the simplest of the three. This method takes time-series FLIM data and applies an intensity (integrated counts) threshold and processes the data through phasor analysis. Intensity thresholding is performed by summing the total counts per pixel over time, applying a minimum count threshold, and setting all pixel values at or below that threshold to zero across each time bin.

The FPA workflow incorporates both intensity thresholding (as defined above) and median filtering. The median filter is applied to the phasor histogram, a common practice in similar applications. A kernel or window size is specified, determining the number of local data points used for filtering. While intensity thresholding is performed before any phasor transformation, the median filter is applied in phasor space and is included as a functionality in both the Python and MATLAB phasor analysis scripts.

The NC-PCA workflow begins by defining the number of principal components for projecting the raw data. For this study, we found that three principal components optimally preserved signal while removing noise. The script then applies the noise-correction (NC) scheme and stores the NC factors for later use. Both the Python and MATLAB scripts also allow tunability in the PCA method selection, depending on dataset size and computational efficiency. For this research, we used Singular Value Decomposition to extract the principal components. Once the dataset was flattened and the principal components were extracted, the original data was projected onto the new basis. The NC factors are then factored into the transformed dataset to properly scale the data back into its original state. Finally, the same intensity thresholding used in TPA and FPA was applied to ensure consistency across all methods. More information on the data analysis methods can be found in the SI.

### Creation of Synthetic FLIM data

A series of synthetic images were first created in PowerPoint. Three distinct images were designed to contain: (1) mitochondria and ribosomes, (2) the nucleus and vacuoles, and (3) the cell wall. When combined, these images mimicked a basic cell structure with distinct geometric features. By initially keeping them separate, we could independently tune the lifetime characteristics associated with each component set. Each image was assigned a lifetime and projected into 256-time bins with signals decaying exponentially according to their specified lifetimes. Once the temporal behavior was modeled, the three time-series datasets were merged into a single dataset.

To ensure that noise in the synthetic data accurately matched experimental data, we applied a Poisson noise distribution. Commonly known as Shot noise, the dominant noise source in FLIM data arises from photon counting errors with a standard deviation proportional to the square root of photon counts. Importantly, time-correlated single-photon detectors count photons independently of time bins. As a result, shot noise is temporally uncorrelated with dependencies only arising from the signal’s exponential decay. To simulate this noise profile, we applied a randomized Poisson distribution to each pixel^30^, setting the mean value to match the corresponding time-bin photon count. Additionally, to prevent zero standard deviation and ensure nonzero noise levels, a mean value of 0.8 (*λ*=0.8 for Poisson distribution) was assigned to background pixels with no fluorescent lifetime. This value was selected based on experimental results (see SI).

Since the signal-to-noise ratio (SNR) of shot noise scales with the square root of photon counts, we varied noise characteristics by adjusting the total photon count per pixel. The total counts per image were chosen to reflect experimental datasets, ranging from 80 to 1,800 photons. This approach ensured that our calculated noise accurately modeled variations encountered in experimental FLIM data while also testing the upper and lower limits of NC-PCA. Additional details on synthetic FLIM dataset construction are provided in the SI.

### Sample Preparation Protocols

#### Coumarin-6 dye

Coumarin-6 (Sigma Aldrich #442631) reconstituted in 200 proof ethanol was used as the calibration standard before each imaging session.

#### Organoid Culture

Patient-derived tumor organoids (PDTOs) were generated from colorectal cancer (CRC) tumor resections received from the USC Norris Comprehensive Cancer Center Translational Pathology Core according to Institutional Review Board (Protocol HS-06–00678) approval. Tissues were processed as outlined previously^43,44^. PDTOs were grown at 37°C and 5% CO_2_ in media which contained supplements as outlined in reference^36^. Additional details are in Table S2.

Sample preparation for FLIM imaging was also done according to previous publications. Briefly, organoids were dissociated into single cells by dissolving BME with Gentle Cell Dissociation Reagent (STEMCELL Technology, Cambridge, MA; 07174) at 4°C with rocking for 30–40 minutes. A P-1000 pipette tip was used to break up the organoid fragments before centrifuging at 300xg for 5 minutes and replacing supernatant with 1:1 PBS:TrypLE (Thermofisher Scientific, Waltham, MA; 12605028 spiked with Y-27632 (STEMCELL Technology, Cambridge, MA; 72302) at 1:1000 dilution. The cell suspension was passed through a 40μm cell strainer before isolating the cell pellet through centrifugation and resuspending in CTO media. 1000 cells/well were plated in a glass-bottom 96 well plate (Mattek, Ashland, MA; P96G-1.5–5-F) and grown in CTO media for 7 days before treatment.

Cells were dosed with various concentrations of Staurosporine (STS; Sigma-Aldrich, St. Louis, MO; 569396), 5-fluorouracil (5-FU; Selleck Chemicals, S1209), 7-ethyl-10-hydroxycamtothecin (SN-38; Sigma-Aldrich, St. Louis, MO; H0165), 3-bromopyruvate (3-BP; Sigma-Aldrich, St. Louis, MO; 16490), or cetuximab (CTX, MedChemExpress, Monmouth Junction, NJ; HY-P9905). Untreated controls for STS, SN-38 and 5-FU were wells of 0.01% DMSO in CTO media. Details on the dosing schedule and quantities are in Table S3. Treatments were applied 72 hours before the first imaging sessions, and imaging was performed. All therapeutic concentrations were created using serial dilution.

### FLIM Imaging Protocols

For all the data presented in the main text, except a portion of the data in Figure S14 and S15, an Olympus FV3000 system equipped with an A320 FastFLIM FLIMbox (ISS Inc., Champaign, IL), hybrid PMT external detectors (Hamamatsu, Shizuoka, Japan), and a Ti:Sapphire 2-photon laser (Spectra-Physics, Mountain View, CA) was used. The second imaging system used to acquire a portion of the data in Figure S14 and S15 was located in a separate imaging facility and was a Zeiss 780 inverted confocal microscope (Carl Zeiss, Jena, Germany). The images were 256 × 256px and were taken with a Plan-Apochromat M27 20X/0.8 NA (Carl Zeiss, Jena, Germany) objective at 12.6 μs pixel dwell time. The PDTO samples were excited with a Ti:Sapphire 2-photon laser (Coherent Chameleon Ultra II) set at 740 nm for NAD(P)H excitation. The emission was split into two channels with a 590 nm long-pass filter, and each channel was further filtered at 460/80 nm for NAD(P)H. External photomultiplier tube detectors (Hamamatsu, Japan R10467U-40) were used to detect the emission for each channel. An A320 FastFLIM FLIMbox (ISS Inc., Champaign, IL.) was used to collect the frequency domain lifetime of NAD(P)H with VistaVision software (ISS Inc., Champaign, IL). Table S4 lists all systems and settings and the corresponding data sets with additional meta-data. For every patient derived organoid presented in the main text, three z-slices with a step-size of 10 μm from the center (20 μm total imaged z-area) were imaged on both systems with a laser power of 15–20 mW.

For Coumarin-6 measurements, we used a 100-frame FLIM image taken as high signal to noise ratio reference data acting as the ground truth. To acquire the low signal to noise ratio Coumarin-6 image, we used 30-frame averaging. Next for both the reference data and raw data we performed a thresholding step to remove pixels with low counts which was followed by a per pixel single exponential fitting. Next the low-frame data (30-frame) was processed using NC-PCA method, and a per pixel exponential fitting was performed on the denoised pixels. The PDTO FLIM dataset in Figure 5, Figure 6 and Figure 7 were originally published in our previous work and is used here for secondary analysis^36^.

The images used as reference data were taken at 100-frame averaging, and the raw images used for denoising were taken at 10-frame averaging.

## Supplementary Material

Supplementary Files

This is a list of supplementary files associated with this preprint. Click to download.

- ScientRepSI.docx

## Figures and Tables

**Table 1.** Co-varied experimental conditions

Treatment	Treatment Concentrations
Staurosporine (STS)	0.01 μM, 1 μM, and 10 μM
5-fluorouracil (5-FU)	0.1 μM, 1 μM, and 10 μM
7-ethyl-10-hydroxycamtothecin (SN-38)	0.01 μM, 0.1 μM, 1 μM, and 10 μM
Cetuximab (CTX)	0.01 μg/mL, 0.1 μg/mL, 1 μg/mL, and 10 μg/mL
3-bromopyruvate (3-BP)	25 μM, 50 μM, 80 μM, and 100 μM
7-ethyl-10-hydroxycamtothecin (SN-38)	0.01 μM, 0.1 μM, 1 μM, 10 μM
Untreated	

## Data Availability

All the data, codes and materials needed for this study are available on Zenodo (https://doi.org/10.5281/zenodo.14895223).
